# Ellagic acid ameliorates aging-induced renal oxidative damage through upregulating SIRT1 and NRF2

**DOI:** 10.1186/s12906-023-03907-y

**Published:** 2023-03-10

**Authors:** Niloufar Naghibi, Asie Sadeghi, Sajjadeh Movahedinia, Mahdis Rahimi Naiini, Mohammad Amin Rajizadeh, Faegheh Bahri, Mahdieh Nazari-Robati

**Affiliations:** 1 Neuroscience Research Center, Institute of Neuropharmacology, Kerman University of Medical Sciences, Kerman, Iran; 2 Department of Clinical Biochemistry, Faculty of Medicine, Kerman University of Medical Sciences, Kerman, Iran; 3 Pathology and Stem Cell Research Center, Department of Pathology, Afzalipour School of Medicine, Kerman University of Medical Sciences, Kerman, Iran; 4 Physiology Research Center, Institute of Basic and Clinical Physiology Sciences, Kerman University of Medical Sciences, Kerman, Iran

**Keywords:** Aging, Kidney, Oxidative stress, Ellagic acid, SIRT1, NRF2

## Abstract

**Background:**

Aging is associated with impaired renal function and structural alterations. Oxidative stress plays a vital role in renal senescence and damage. Sirtuin 1 (SIRT1) is thought to protect cells from oxidative stress through nuclear factor erythroid 2-related factor 2 (NRF2). Ellagic acid (EA), a natural antioxidant, has been demonstrated to have renoprotective roles in vitro and in vivo. This study investigated if SIRT1 and NRF2 mediate the protective effects of EA in aged kidneys.

**Methods:**

Male Wistar rats were divided into three groups including young (4 months), old, and old + EA (25 months). Young and old groups received EA solvent, while the old + EA group was treated with EA (30 mg/kg) by gavage for 30 days. Then, the level of renal oxidative stress, SIRT1 and NRF2 expression, kidney function parameters, and histopathological indices were measured.

**Results:**

Treatment with EA significantly increased the level of antioxidant enzymes and reduced malondialdehyde concentration (*P* < 0.01). Moreover, EA administration remarkably upregulated mRNA and protein levels of SIRT1 and NRF2 as well as deacetylated NRF2 protein (*P* < 0.05). Additionally, EA treated rats improved kidney function and histopathological scores (*P* < 0.05).

**Conclusions:**

These findings suggest that ellagic acid exerts protective effects on aged kidneys by activating SIRT1 and NRF2 signaling.

**Supplementary Information:**

The online version contains supplementary material available at 10.1186/s12906-023-03907-y.

## Background

Aging is a physiological process associated with irreversible functional impairment and predisposition to chronic diseases. As with other organ systems, the functional capabilities of the kidney decline progressively with aging, which primarily manifests as reductions in the glomerular filtration rate (GFR) [1]. On the structural level, the kidney experiences several changes, including nephrosclerosis, glomerular basement membrane thickening, and accumulation of extracellular matrix with aging. These alterations impair the ability of the kidney to recover from injury, thus resulting in the increased susceptibility of the aged kidneys to acute kidney injury (AKI) and the development of progressive chronic kidney disease (CKD) [2].

The aging process is closely connected to enhanced mitochondrial dysfunction and reduced antioxidant capacity leading to redox signaling disruption and a wide range of phenotypic changes, including altered gene expression, arrested cell proliferation and growth, and cellular senescence [3, 4]. In addition, the reduction in mitochondrial function during aging results in a decrease in cellular NAD^+^ levels, which would be expected to compromise the activities of NAD^+^-dependent enzymes, including protein deacetylases of the Sirtuin (SIRT) family [5]. In mammals, seven sirtuins constitute an evolutionarily conserved group of enzymes involved in various interrelated cellular processes, such as metabolism, mitochondrial biogenesis, autophagy, and apoptosis [6]. SIRT1 is the most well-studied member of the sirtuin family in the kidney, which is widely expressed in tubular cells and podocytes [7]. By targeting various transcriptional factors for deacetylation, SIRT1 provides renoprotections by reducing interstitial fibrosis, inhibiting tubular and glomerular cell apoptosis, suppressing inflammation, and improving mitochondrial function, and regulating blood pressure [8]. Therefore, SIRT1 dysfunction associated with aging may contribute to the initiation and progression of age-related kidney diseases [6].

Nuclear factor erythroid 2-related factor 2 (NRF2) is a crucial regulator of oxidative stress response that induces transcription of target genes encoding proteins associated with redox regulation through binding to antioxidant response element (ARE) in ARE-driven gene promoters [9]. In normal conditions, NRF2 is thought to be sequestered by Kelch-like ECH-associated protein 1 (Keap1) in the cytosol. Still, upon a redox imbalance, NRF2 dissociates from Keap1 and translocates to the nucleus to initiate the transcription of genes involved in antioxidant defense. However, NRF2 has been described to be dysfunctional during aging, resulting in impaired oxidative stress response signaling [10]. Multiple posttranslational modifications including acetylation-deacetylation regulate the function of NRF2. Consistent with the view that NRF2 is positively regulated by deacetylation, SIRT1 has been described to deacetylate NRF2, which increases the stability and transcriptional activity of NRF2, thus improves the resistance of cells to oxidative damage [11, 12]. In addition, recent findings have shown that SIRT1 can protect cells from oxidative stress by upregulating the level of NRF2 protein [13]. Therefore, pharmacological targeting of SIRT1 in the kidney may counteract the pathological changes involved in kidney aging.

Ellagic acid (EA, 2,3,7,8-tetrahydroxybenzopyrano (5,4,3-cde) benzopyrano-5,10-dione), a naturally occurring polyphenolic compound, is commonly found in nuts, vegetables, and fruits, such as pomegranate, raspberries, strawberries, and grapes. It is well established that EA has a wide range of biological activities, including antioxidant, anti-inflammatory, anticancer, and antidiabetic properties [14]. Recent findings have suggested that EA ameliorates oxidative stress through the upregulation of NRF2 and, thus, the induction of antioxidant enzymes [15]. Furthermore, EA has been shown to protect kidneys against carbon tetrachloride-induced oxidative damage via activation of NRF2-driven antioxidant signal pathway [16]. In addition, EA has recently been reported to prevent iron oxide-induced nephrotoxicity by inducing the expression of SIRT1 in renal tissues [17]. Despite these beneficial effects of EA, the role of EA in the aging kidney has not been well defined. Therefore, in this study, we aimed to investigate the possible effect of EA on renal SIRT1 and NRF2 and kidney function in aged rats.

## Materials and methods

### Chemicals and reagents

All antibodies used in this research were purchased from Santa Cruz Biotechnology company (CA, USA). Chemicals were provided by Sigma-Aldrich company (MO, USA). The origin of other reagents and kits has been described in experimental methods.

### Animals and experimental design

In this experimental study, a total of 21 male Wistar rats including 14 aged (25-month-old) and 7 young (4-month-old) rats were used. Animals were housed in standard cages (2–3 rats per cage) under controlled environmental conditions (22 ± 1 °C; 60% humidity, and 12 h light/dark cycle) with free access to standard food and tap water. All experiments were approved by the Ethics Committee of Kerman University of Medical Sciences (IR.KMU.AH.REC.1400.041), and performed according to the guide for the care and use of laboratory animals by National Institutes of Health (NIH). Old animals were randomly divided into two groups of seven rats. EA (Sigma–Aldrich, USA) was dissolved in saline containing 0.1% DMSO. Then young and aged control rats received EA solvent by gavage, while the rats in EA group were treated with EA (30 mg/kg). All treatments were given once daily and continued up to 30 days. The dose of EA was selected based on previous studies [18, 19].

### Sample collection

At the end of experimental period, rats were placed in individual metabolic cages and 24 h urine was collected to measure albumin and creatinine concentration. Then, animals were deeply anesthetized with ketamine (50 mg/kg) and xylazine (5 mg/kg) and blood samples were collected via cardiac puncture for urea and creatinine measurement. Kidneys were immediately taken and washed with cold isotonic saline. The left kidney was fixed in 10% formalin for histopathological examinations and the right kidney stored at -80℃ for biochemical analysis.

### Assessment of renal oxidative stress level

The concentration of malondialdehyde (MDA) as a marker of oxidative stress and the levels of total antioxidant capacity (TAC), catalase (CAT) and superoxide dismutase (SOD) activity were measured using commercial kits and according to the manufacturer’s protocol (Kiazist, Iran). Briefly, kidney tissues were homogenized in lysis buffer containing protease inhibitors (Sigma–Aldrich, USA). After centrifugation by a 3-18KS Sigma centrifuge (Sigma, Germany), supernatants were collected for next analysis. MDA level was quantified by measuring thiobarbituric acid reactive substances produced in the reaction of MDA with thiobarbituric acid. TAC level was measured based on the capacity to convert Cu^2+^ to Cu^+^ ion. The activity of catalase was determined according to the reaction of the enzyme with methanol in the presence of hydrogen peroxide and measurement of generated formaldehyde. SOD activity was assayed by measuring the dismutation of superoxide radicals generated by the xanthine/xanthine oxidase system. Protein concentration of lysates was measured using Bradford method. Then the levels of oxidative stress markers were normalized to protein content [20, 21].

### Detection of acetylated NRF2

The kidney tissue was lysed in ice-cold lysis buffer (50 mM Tris, 150 mM NaCl, 1% NP-40) (1:5 w/v) in the presence of protease inhibitor. The lysate was centrifuged at 12,000 rpm for 15 min at 4℃. Then the supernatant was immunoprecipitated with antibody specific to NRF2 (sc-33649, 1:50) at 4℃ overnight. The immunocomplexes were then collected and subjected to western blotting using anti-acetyl lysine antibody (sc-9441, 1:700).

### Western blot analysis

The kidney tissue was homogenized in ice-cold RIPA buffer (1:10 w/v) containing protease inhibitor. The homogenate was centrifuged at 12,000 rpm for 15 min at 4℃. Then the resulting supernatant was collected. Protein concentration was determined by bicinchoninic acid assay. Equal amounts of extracted proteins were separated by 10% sodium dodecyl sulfate–polyacrylamide gel electrophoresis (SDS-PAGE) and transferred onto the PVDF membranes. The membranes were blocked with 5% non-fat milk in TBST buffer at 4℃ overnight. Then the membranes were incubated with primary antibodies specific to SIRT1 (sc-74465, 1:500), NRF2 (sc-365949, 1:500), and β-actin (sc-47778, 1:1000) for 1 h at room temperature. After washing in TBST, membranes were incubated with horseradish peroxidase (HRP)-conjugated secondary antibodies (sc-2357, 1:10,000) for 1 h at room temperature. Then membranes were washed in TBST and detected with ECL kit (Bio-Rad, USA) for protein bands. The detected bands were quantified using ImageJ analyzing software. β-actin was used as an internal reference.

### RNA extraction and Real-time PCR

Total RNA was extracted from kidney samples using Trizol reagent (GeneAll Biotechnology, Korea) according to the manufacturer’s instructions. The concentration and purity of RNA were determined by a NanoDrop ND-1000 spectrophotometer (Thermo Scientific, USA). Then complementary DNA (cDNA) was synthesized according to the protocol of cDNA synthesis kit (Parstous, Iran). Real-time PCR amplification was performed on Mic PCR system (BMS, Australia) using SYBR Green master mix (Ampliqon, Denmark), cDNA and primers. Primer sequences were as follows: *SIRT1*, 5′-GGTAGTTCCTCGGTGTCCT -3′ (forward) and 5′-ACCCAATAACAATGAGGAGGTC-3′ (reverse); *NRF2*, 5′-CACATCCAGACAGACACCAGT-3′ (forward) and 5′-CTACAAATGGGAATGTCTCTGC-3′ (reverse); *GAPDH*, 5′-AGGTTGTCTCCTGTGACTTC -3′ (forward) and 5′-CTGTTGCTGTAGCCATATTC-3′ (reverse). PCR conditions were 95℃ for 10 min followed by 40 cycles of 95 °C for 40 s, 62 °C for 25 s, and 72 °C for 15 s. Relative gene expression was calculated using ΔΔCT method. *GAPDH* gene was used as internal control [20].

### Measurement of renal function

Blood urea and creatinine levels were analyzed by commercial kits (Pars Azmun, Iran). Urinary concentration of creatinine was also measured. Urine albumin was determined by immunoturbidometric kit (BioSystems, Spain). To estimate GFR, creatinine clearance was calculated using the standard formula: [urine creatinine (mg/dl) × urine volume (ml/24 h)] /[serum creatinine (mg/dl) × 1440 (min/24 h)].

### Histopathological Examination

Fixed kidney tissues in 10% formalin were embedded in paraffin and cut into 4-μm sections. Then renal sections were stained with hematoxylin and eosin (H&E). Moreover, to detect collagen fibers or fibrosis, Masson’s trichrome staining was performed. For each section, five fields were observed under a CX41 Olympus light microscope and digitally photographed (Olympus, Japan). Necrosis, inflammation, tubular atrophy, tubulointerstitial fibrosis were then analyzed and scored as described previously [20].

### Statistical analysis

All data were presented as mean ± SD. SPSS software 20.0 (IBM, USA) were employed to perform data analysis. Statistical significance was determined using Mann–Whitney with the level of significance set at *P* = 0.05.

## Results

### Alleviation of renal oxidative stress after treatment with EA

**Fig. 1:**
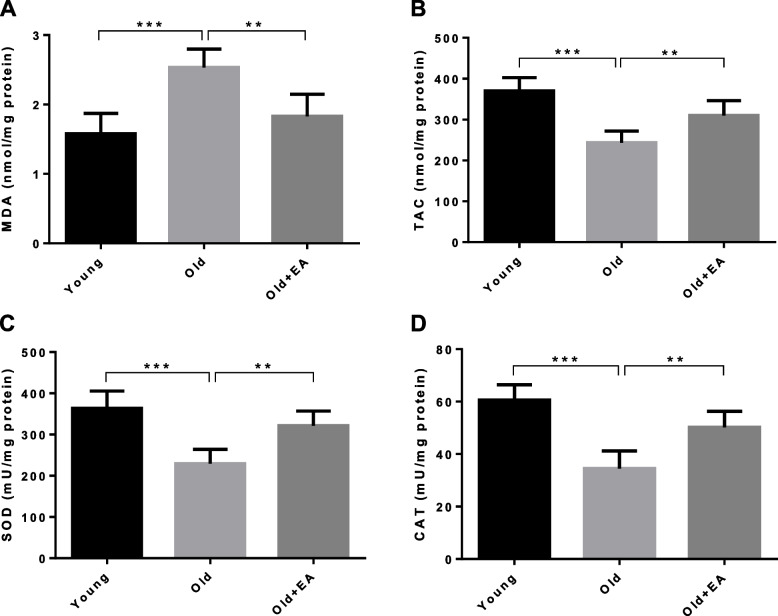
Effect of ellagic acid (EA) on renal level of **A** malondialdehyde (MDA) **B** total antioxidant capacity (TAC) **C** superoxide dismutase (SOD) and **D** catalase (CAT) in different groups (*n* = 7). All data are mean ± SD. Statistical significance expressed as ** *p<*0.01, *** *p<*0.001

The obtained results in Fig. 1 showed a marked increase in the level of MDA and a significant decline in the levels of TAC, SOD and CAT in renal tissues of aged rats compared with those of young animals (*P* < 0.001). However, administration of EA could attenuate oxidative stress in senescent kidneys, evidenced by a significant reduction in the level of MDA and a remarkable elevation in the levels of TAC, SOD and CAT in comparison with untreated aged renal tissues (*P* < 0.01).

### Upregulation of SIRT1 and NRF2 at mRNA level after treatment with EA

**Fig. 2:**
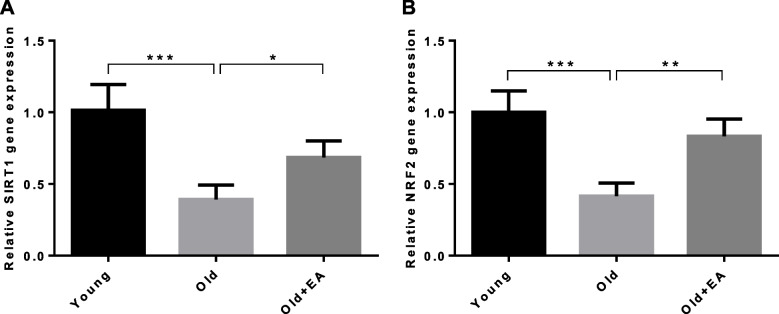
Effect of ellagic acid (EA) on renal level of **A** *SIRT1* and **B** *NRF2* mRNA transcripts in different groups (*n* = 7). All data are mean ± SD. Statistical significance expressed as * *p<*0.05, ** *p<*0.01, *** *p<*0.001

Results indicated a significant reduction in the level of *SITR1* and *NRF2* gene expression in the kidneys of old compared with young rats (*P* < 0.001). However, the level of *SIRT1* and *NRF2* mRNA was upregulated by 1.7-fold and twofold, respectively in aged animals following treatment with EA compared with old control rats (*P* < 0.05 and *P* < 0.01, respectively) (Fig. 2).

### Alterations of SIRT1, NRF2 and acetylated NRF2 at protein level after treatment with EA

**Fig. 3:**
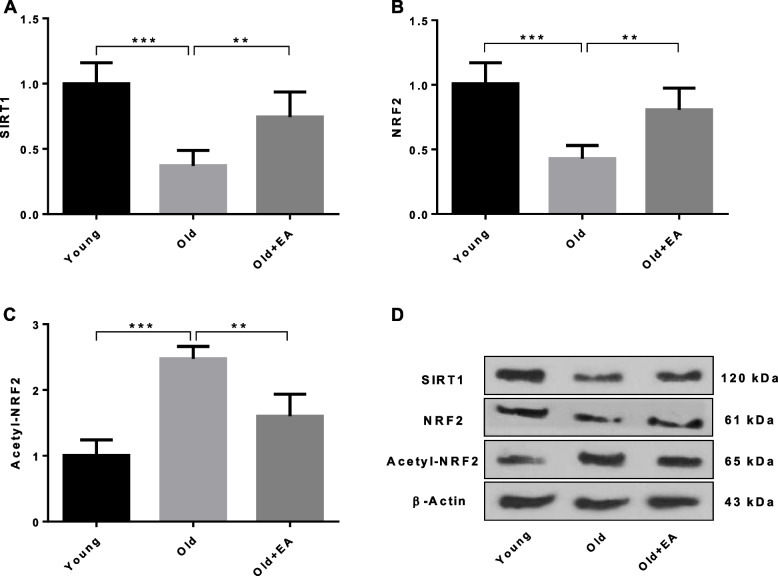
Effect of ellagic acid (EA) on renal level of **A** SIRT1, **B** NRF2 and **C** Acetyl-NRF2 proteins in different groups (*n* = 7). **D** Representative immunoblot images of SIRT1, NRF2, Acetyl-NRF2 and β-Actin. All data are mean ± SD. Statistical significance expressed as ** *p<*0.01, *** *p<*0.001

Western blot analysis of SIRT1 and NRF2 revealed a significant decrease in the level of these proteins in renal tissues of aged rats compared with young animals (*P* < 0.001). Conversely, old rats showed a significant elevated level of acetylated NRF2 (Ac-NRF2) in comparison with young group (*P* < 0.001). However, SIRT1 and NRF2 proteins were significantly increased in the kidneys of senescent rats treated with EA compared with aged control group (*P* < 0.01). In contrast, there was a significant reduction in the level of Ac-NRF2 in the renal tissues of aged rats received EA in comparison to those of old control animals (*P* < 0.01) (Fig. 3).

### Amelioration of renal function after treatment with EA

Old rats showed significant elevated serum levels of urea (*P* < 0.001) and creatinine (*P* < 0.01) as well as increased urine level of albumin compared with young animals (*P* < 0.001). Additionally, creatinine clearance rate was decreased remarkably with aging (*P* < 0.001). Comparing with senescent control group, EA administration reduced serum urea (*P* < 0.05) and creatinine (*P* < 0.01) and improved creatinine clearance rate significantly (*P* < 0.05). However, urinary albumin excretion did not change after treatment with EA (*P* > 0.05) (Table 1).

**Table 1:** Kidney function parameters in different groups

Parameter	Young	Old	Old + EA
**Urea (mg/dl)**	54.1 ± 5.2	68.5 ± 5.8^***^	59.2 ± 4.7^#^
**Creatinine (mg/dl)**	0.64 ± 0.066	0.82 ± 0.075^**^	0.67 ± 0.054^##^
**Creatinine clearance rate (ml/min/kg)**	2.5 ± 0.30	1.5 ± 0.25^***^	2.0 ± 0.27^#^
**Albuminuria (μg/day)**	13.9 ± 2.5	20.2 ± 2.9^***^	17.8 ± 2.1
**Body weight (g)**	329 ± 13	400 ± 15^***^	414 ± 17
**Kidney weight (g)**	1.0 ± 0.13	1.4 ± 0.17^***^	1.3 ± 0.14

Data are presented as mean ± SD for *n* = 7 rats in each group

*EA* Ellagic acid

^**^ *p<*0.01 vs young control

^***^ *p<*0.001 vs young control

^#^ *p<*0.05 vs old control

^##^ *p<*0.01 vs old control

### Alterations of histopathological scores after treatment with EA

**Fig. 4:**
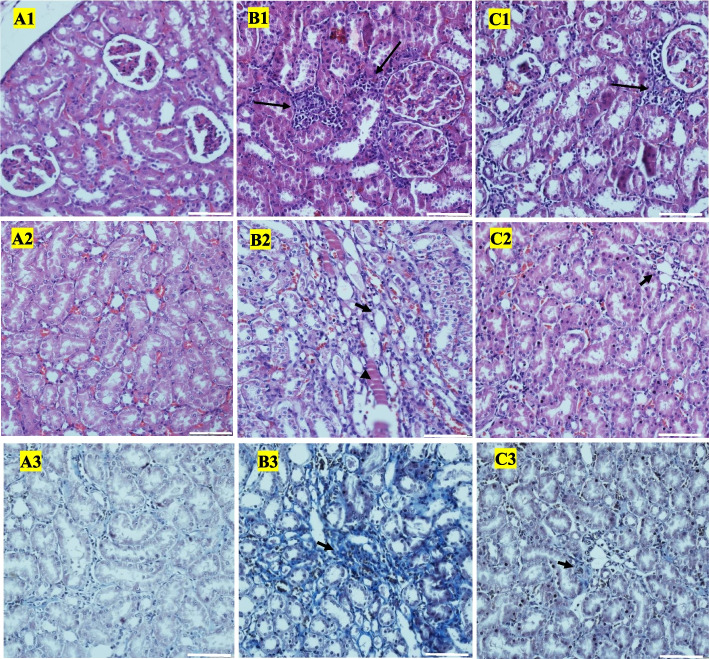
Histopatholoy of kidney tissue in different studied rat groups. Representative images of Hematoxylin and Eosin (original magnification × 200) stain of (1) cortical and (2) medullary areas and (3) Trichrome stain (original magnification × 200) are shown (scale bars = 100 μm). **A** Young control rats renal tissue with absent to minimal inflammation, no atrophy or fibrosis; **B** Old control rats kidney tissue exhibiting marked inflammation (long arrow), evidence of hyalin cast formation (arrow head), and mild tubulointerstitial atrophy and fibrosis (short arrow). **C** Treated old rats renal tissue with mild inflammation (long arrow), minimal atrophy and fibrosis (short arrow)

Histopathologically, renal sections of aged rats displayed elevated levels of inflammation, tubular atrophy and fibrosis compared with those of young animals (*P* < 0.01) (Fig. 4). However, treatment with EA caused significant improvement in these histopathological features in comparison with senescent control tissues (*P* < 0.05) (Table 2).

**Table 2:** Grading of histopathological features in different groups

Histopathological feature	Young	Old	Old + EA
**Inflammation**	0.0 ± 0.0	3.0 ± 0.4^**^	2.2 ± 0.3^#^
**Necrosis (%)**	0.0 ± 0.0	0.0 ± 0.0	0.0 ± 0.0
**Tubular atrophy (%)**	0.0 ± 0.0	14.0 ± 2.4^***^	4.0 ± 1.0^##^
**Tubulointerstitial fibrosis (%)**	0.0 ± 0.0	10.4 ± 2.8^**^	2.6 ± 0.8^#^

Pathological changes were scored as described in Materials and Methods. Data are presented as mean ± SD for *n* = 7 rats in each group

*EA* Ellagic acid

^**^ *p<*0.01 vs young control

^***^ *p<*0.001 vs young control

^#^ *p<*0.05 vs old control

^##^ *p<*0.01 vs old control

## Discussion

Renal aging is associated with disruptions in cellular homeostasis, leading to a reduction in major protective factors such as SIRT1 and a decrease in responsiveness to physiological damage, including oxidative stress [22]. Therefore, targeting the SIRT1 signaling pathway may be a potential strategy to prevent or slow kidney aging. In the current study, we showed that treatment with ellagic acid (EA) could upregulate the expression of SIRT1 and ameliorate oxidative stress through induction of NRF2 expression and deacetylation. Moreover, EA administration could attenuate aging-induced renal dysfunction and lesions.

Increased oxidative stress and mitochondrial dysfunction are believed to be significant factors contributing to aging [5]. Mitochondria are the main producer of ROS in cells. However, a significant reduction occurs in electron flow through the mitochondrial respiratory chain with aging, which favors increased ROS generation. Therefore, senescent cells have a high content of ROS and accumulative oxidative damage to biomolecules, particularly DNA and Proteins [23]. The removal of oxidative DNA damage depletes intracellular NAD^+^ pools and impairs the activity of NAD^+^-dependent enzymes, such as sirtuins [5].

SIRT1, the most extensively studied sirtuin, has been increasingly recognized to play various roles in gene silencing, stress resistance, apoptosis, inflammation, and aging [24]. Emerging evidence shows that SIRT1 expression decreases with aging and may be involved in age-associated diseases [25]. Consistent with our data, Kwon et al. reported that SIRT1 expression was reduced in the kidney of 24-month-old mice compared with 6-month-old mice [26]. In a similar study, SIRT1 expression was indicated to be lower in the renal tissues of 24-month-old mice compared to 2 and 12-month-old mice [27]. However, in the present study, EA administration was found to augment the level of SIRT1 at both gene and protein expression in aged kidneys. EA, a natural polyphenolic compound, has received considerable attention because of its various biological properties, such as radical scavenging, anti-inflammatory, antiviral, cancer, and diabetes-prevention activities [14]. A recent study reported that EA administration significantly enhanced the expression of SIRT1 in rat kidneys and ameliorated cisplatin-induced nephrotoxicity [28]. Additionally, EA has been shown to protect renal tissues against iron oxide-induced damage by promoting the expression of SIRT1 [17]. These findings suggested that SIRT1 plays a crucial role in the protective effects of EA on renal tissue damage.

NRF2 is a redox-sensitive transcription factor that regulates the basal expression of antioxidant genes and confers cytoprotection against oxidative stress. In normal conditions, Keap1, a cysteine-rich protein, is associated with NRF2. Exposure to reactive oxidants leads to the oxidation of critical cysteine residues, thus resulting in NRF2 release and translocation to the nucleus, where it binds to DNA-responsive elements and activates the transcription of several antioxidant genes and major ROS scavenging enzymes. It is well established that aging is associated with a gradual decline in NRF2 level and pathway responsiveness, promoting oxidative injury in senescence tissues [9, 29]. In this sense, the results of the current study indicated a redox imbalance in aged kidneys as evidenced by the downregulation of NRF2 and diminished levels of SOD, CAT, and TAC and elevated levels of MDA. Similar features of oxidative damage associated with reduced NRF2 were previously observed in the kidney of 24-month-old rats [30]. The renal protective effect of NRF2 is supported by the fact that NRF2 gene ablation increased renal oxidative stress and inflammation in the experimental model of diabetes [31].

Moreover, NRF2 knockout mice exhibited more severe kidney injury during ischemic and nephrotoxic insults than wild-type mice [32]. In contrast, pharmacological interventions using NRF2 activators attenuated markers of kidney damage from oxidative stress in various experimental models [33]. In the current study, we found that EA administration reversed the downregulation of NRF2 and subsequent oxidative stress induced by aging in the kidney tissues, suggesting EA potential effect in activating the NRF2 signaling pathway in aged kidneys. In this context, a recent study reported that EA could markedly prevent kidney damage against carbon tetrachloride-induced oxidative stress through the upregulation of NRF2 [16]. Moreover, EA was confirmed to protect against diabetic nephropathy by modulating the transcription and activity of NRF2 [34].

Activation of the NRF2 signaling pathway is the primary mechanism to combat oxidative stress. Although NRF2 activity is mainly controlled by Keap1, other forms of regulation of NRF2 function include acetylation-deacetylation of NRF2 [35]. In the present study, we found that EA administration increased SIRT1-mediated deacetylation of NRF2 in senescent renal tissues. In this regard, a recent investigation reported that SIRT1 exerts its antioxidant activity by promoting nuclear translocation, DNA binding, transcriptional activity, and target genes expression of NRF2 in a deacetylase-dependent manner [13]. Additionally, acetylation of NRF2 was shown to reduce NRF2 stability and impaired antioxidant defenses. Therefore, SIRT1-mediated deacetylation of NRF2 was proposed to increase NRF2 stability and enhance antioxidant gene expression [36, 37]. In line with our results, the administration of resveratrol to aged mice was demonstrated to upregulate the expression of SIRT1 and NRF2, leading to reduced renal oxidative damage and dysfunction [38]. Moreover, activation of SIRT1 in the kidneys of diabetic mice could elevate NRF2 antioxidant signaling and provide remarkable protection against diabetic nephropathy-induced renal oxidative stress [11]. In contrast, the depletion of SIRT1 was associated with a reduction in the transcriptional activity of NRF2, indicating that SIRT1 promoted the activation of NRF2 signaling pathway [39].

Aging is characterized by progressive structural and functional deterioration of kidneys. With aging, many subjects exhibit reductions in glomerular filtration rate (GFR) and renal blood flow (RBF), which occur in concert with a decline in renal mass, tubulointerstitial fibrosis, and increased glomerulosclerosis [2]. Our results revealed impaired renal function in aged rats as indicated by reduced levels of GFR and increased levels of serum urea and creatinine, and albuminuria levels. Histologically, in the present study, senescent renal tissues displayed elevated levels of inflammation, tubular atrophy, and fibrosis, which agree with the findings of a previous study [27]. Increased oxidative stress is considered to be the main pathogenic factor underlying these features and contributing to the elevated oxidative stress; reduced levels of SIRT1 and NRF2 in aged kidneys have been suggested [2, 9]. In this regard, a recent investigation demonstrated that mice with catalytically inactive SIRT1 had a lower glomerular numbers and GFR [40].

Additionally, the diminishment of NRF2 antioxidant capacity aggravates renal tubular apoptosis and atrophy as well as interstitial fibrosis under oxidative stress conditions [41]. However, pharmacological intervention using SIRT1 and NRF2 activator resveratrol improved renal function, proteinuria, glomerulosclerosis, tubular fibrosis, and inflammation in aged kidneys [38, 42]. Here, we showed that EA administration ameliorated age-associated renal dysfunction and histopathological alterations, which is thought to be mediated through SIRT1. Similar findings of improved renal function and histopathological features mediated by SIRT1 were also reported in several rat models of nephrotoxicity [17, 28].

The findings of this study demonstrated that EA has nephroprotective effects through SIRT1/NRF2 pathway in the aging kidneys. However, the reverse experiment was not performed through SIRT1 inhibition to provide more evidence which is one limitation of this study. Therefore, additional experiments using pharmacological inhibitors are required to confirm our conclusion further. Another limitation of our study is that we did not determine NRF2 nuclear translocation and activity, which can be explored in future studies.

## Conclusion

Taken together, our results provided evidence that EA administration upregulated SIRT1 and NRF2 in aged kidneys. SIRT1 activation further promoted NRF2 deacetylation and subsequent activation, leading to increased antioxidant levels in senescent renal tissues. Moreover, treatment with EA improved renal function and ameliorated histopathological features in aged kidneys. These findings provided an experimental basis for the application of EA to delay the process of aging in kidneys. However, additional clinical studies for EA safety are required.

## Supplementary Information

**Additional file 1.**

## Data Availability

All data generated or analyzed during this study are included in this published article.

## Abbreviations

AKI: Acute kidney injury
ARE: Antioxidant response element
CAT: Catalase
CKD: Chronic kidney disease
EA: Ellagic acid
GFR: Glomerular filtration rate
Keap1: Kelch-like ECH-associated protein 1
MDA: Malondialdehyde
NRF2: Nuclear factor erythroid 2-related factor 2
SIRT1: Sirtuin 1
SOD: Superoxide dismutase
TAC: Total antioxidant capacity
