# Supplementary material for: Ellagic acid ameliorates aging-induced renal oxidative damage through upregulating SIRT1 and NRF2
Source: BMC Complement Med Ther. 2023 Mar 10;23:77. doi: 10.1186/s12906-023-03907-y (PMC9999491; doi:10.1186/s12906-023-03907-y)
Supplement: Supplementary file 1 — Additional file 1. [file 12906_2023_3907_MOESM1_ESM.pdf]

Figure 3

D

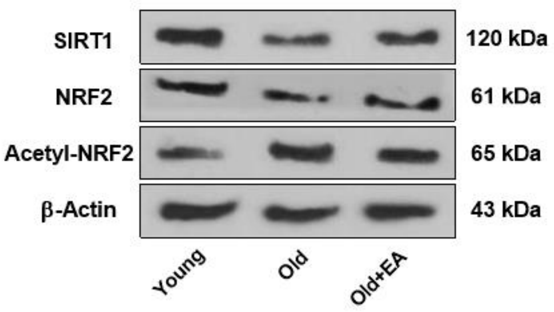

SIRT1 (120 kDa)

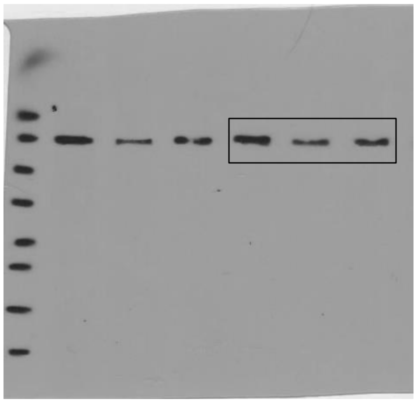

Loading order: Marker, Young 1, Old 1, Old+EA 1, Young 2, Old 2, Old+EA 2

NRF2 (61 kDa)

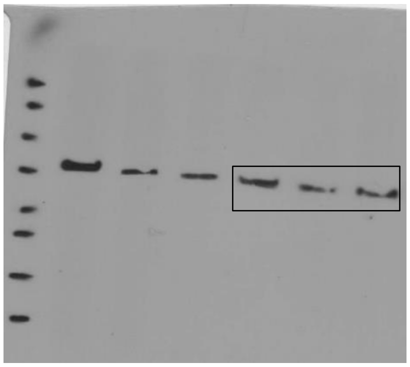

Loading order: Marker, Young 1, Old 1, Old+EA 1, Young 2, Old 2, Old+EA 2

Acetyl-NRF2 (65 kDa)

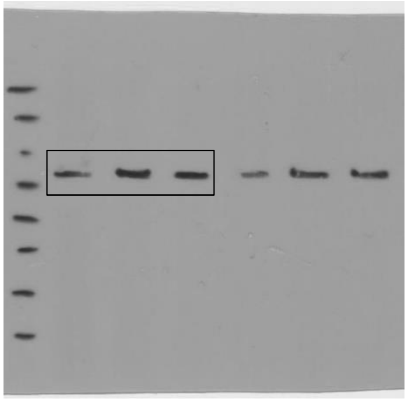

Loading order: Marker, Young 1, Old 1, Old+EA 1, Young 2, Old 2, Old+EA 2

β-Actin (43 kDa)

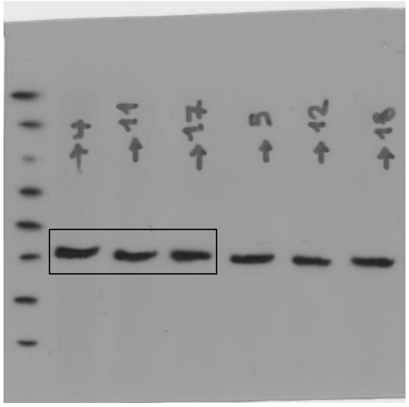

Loading order: Marker, Young 1, Old 1, Old+EA 1, Young 2, Old 2, Old+EA 2
